# A Rare Case of Metronidazole Overdose Causing Ventricular Fibrillation

**DOI:** 10.7759/cureus.24728

**Published:** 2022-05-04

**Authors:** Mohamed Abdelgadir M Elgassim, Amin Saied Sanosi Saied, Moayad A Mustafa, Amro Abdelrahman, Ibtesam AlJaufi, Waleed Salem

**Affiliations:** 1 Emergency Medicine, Hamad General Hospital, Doha, QAT; 2 Internal Medicine, Hamad General Hospital, Doha, QAT; 3 Internal Medicine, Taylor's University Lakeside Campus, Subang Jaya, MYS; 4 Emergency Department, Hamad Medical Corporation, Doha, QAT

**Keywords:** adverse reaction, new drug reaction, ventrilcular fibrillation, ventricular fibrillation (vf) storm, metronidazole

## Abstract

Ventricular fibrillation is not known as a complication of metronidazole poisoning. Although some arrhythmias have been reported as a complication of metronidazole intake while taking antiarrhythmic medications, most such arrhythmias are possibly related to co-ingestion of drugs with metronidazole as it affects the metabolism of these drugs. In this case, ventricular fibrillation occurred in a young patient without preexisting medical conditions or any other known drug ingestion, which was never been reported before.

We present a case of an 18-year-old male brought in by the ambulance service after attempting to end his life by overdosing on metronidazole. While being transported he developed ventricular fibrillation and received an electric shock, which reverted the episode. Laboratory investigations did not show any clear cause that might have precipitated his arrhythmia.

## Introduction

Metronidazole is a nitroimidazole antibiotic that is used in the treatment of a wide spectrum of microbial infections. Although a generally safe drug that is widely dispersed, it has common side effects that include: nausea, vomiting, diarrhea, and hypoglycemia. In cases of severe overdoses, it can cause some cardiac arrhythmias among other life-threatening complications. A common culprit of these cardiac complications is the co-ingestion of metronidazole with another medication that can cause these complications when present in excess. That is due to metronidazole interfering with the other medications metabolism leading to its accumulation in the bloodstream.

To the best of our knowledge this is the first ever reported case of ventricular fibrillation secondary solely to metronidazole overdose.

## Case presentation

Background

We report the case of an 18-year-old man with no underlying chronic medical illness history, previous gastric sleeve surgery three years ago, recent presentation for dumping syndrome 14 days ago, currently in cadet military training camp. Reports of depression in the military training and previous attempts to flee through medical diagnoses were found. His family history included suicide by his sister at the age of 20 years through a drug overdose.

Presentation

**Figure 1:**
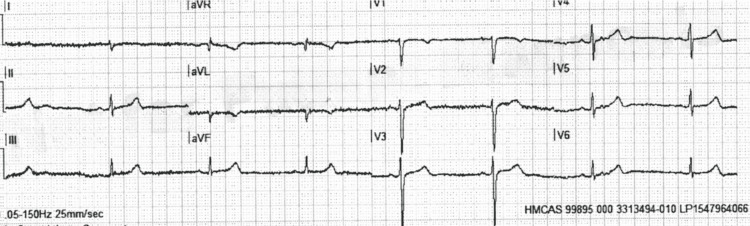
Initial 12 lead ECG reading taken at 1858 hours.

The patient was presented as a case of suicide attempt through the means of metronidazole overdose. He had swallowed approximately 30 tablets of 200 mg metronidazole in the morning at around 0900 hours, denying co-ingestion. He self-presented to the military clinic as a case of poisoning immediately after and was complaining of epigastric pain and nausea and collapsed afterward. The military clinic physician contacted the ambulance service at 1805 hours as a case of poisoning and suicide attempt. He was found by our Ambulance Medical Services (AMS) service in the military clinic bed at 1848 hours, lying unconsciously, with no monitoring, and no IV lines attached. Initial assessment by the AMS service showed an unconscious patient with a patent airway maintained, breathing at 18 breaths per minute with good equal bilateral air entry, slow pulses with sinus bradycardia at 48 bpm (patient’s baseline was 70-90 bpm) (ECG is shown in Figure 1), and blood pressure was maintained at 138/78 mmHg. He had cold peripheries, with normal capillary refilling time. He was nonresponsive to stimuli, pupils were 3 mm bilaterally and sluggishly reactive, with a Glasgow Coma Scale of 3 (E1V1M1). His recorded temperature was 35.4 degrees Celsius.

**Figure 2:**
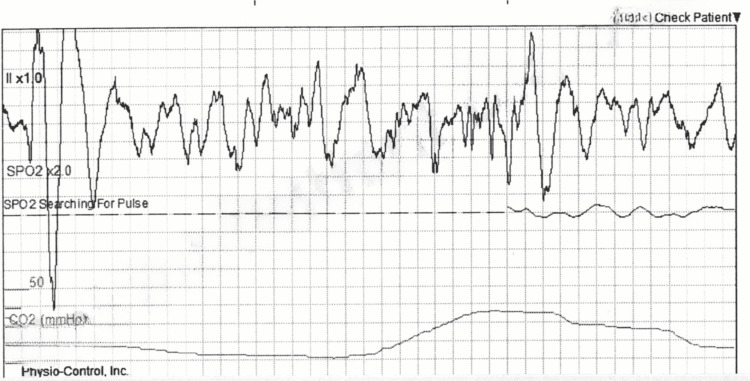
ECG showing readings consistent with ventricular fibrillation at 1911 hours.

**Figure 3:**
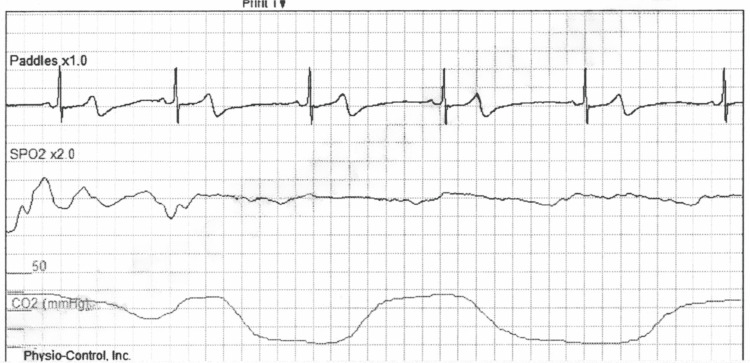
Post-defibrillation ECG reading showing return to sinus bradycardia taken at 1929 hours.

While the patient was being transported to the emergency department under AMS monitoring at 1911 hours, his ECG reading showed signs consistent with ventricular fibrillation as shown in Figure 2. 200J energy shock was delivered at that point and a return of spontaneous circulation (ROSC) was achieved. The patient regained consciousness at 1913 hours, with a full GCS score of 15, and a heart rate of 63 bpm. Next, an ECG reading was taken at 1929 hours showing the return of sinus bradycardia with QT prolongation as shown in Figure 3. The patient maintained consciousness until his arrival at the hospital at 1955 hours.

Upon arrival at the emergency department, the patient was vitally stable except for a random blood glucose reading of 2.5 mmol, which was corrected through oral juice ingestion to 6.8 mmol. He maintained normal readings for his vitals and ECG readings throughout his entire stay in the emergency department. The patient was expressing suicidal ideation and was placed on high risk for suicide alert; however, psychiatry services assessed him and suggested that secondary gain is the main purpose of ingestion and removed him from the high-risk alert before signing off. The toxicology team assessed the patient and recommended close monitoring for common side effects of metronidazole overdose while at the same time investigating other causes for the ventricular fibrillation episode. Cardiology assessed the patient and proceeded to do a full workup including two sets of troponins and echocardiography, which both came back within normal range. The patient’s entire workup including toxicology screening for other co-ingestions and CT head showed no abnormalities, except for slightly elevated aspartate aminotransferase (AST) at 78 U/L and alanine transaminase (ALT) at 44 U/L, compared to his previously normal readings when he presented two weeks ago. His electrolyte levels were within the normal range. The patient was also assessed by the medical ICU (MICU) on-call at which point the story the patient narrated had changed, and he insisted that did not attempt to end his life, but rather was having epigastric pain the night before and ingested only two tablets of metronidazole. He reported that he was only sleepy when the AMS service delivered his shock; however, he doesn’t fully recall the episode but remembers feeling like he was punched in the chest. The patient was scheduled to be started on Holter monitoring by cardiology in the morning; however, before he was started on Holter monitor at 0845 hours, the patient absconded from the hospital, without notification.

## Discussion

Metronidazole is a nitroimidazole antibiotic that is used to treat a wide array of protozoans and anaerobic bacteria. This group of antibiotics works by reductions of the nitro group to protonated nitro electron radical anion, in order to oxidize DNA, hence strand break strands to kill susceptible cells [1].

The range of use of metronidazole ranges from being used in the treatment of Crohn’s disease with infectious complications to being used in the regimen used to treat gastric and pancreatic ulcers caused by *Helicobacter pylori* [2]. The advantages of metronidazole are that the percentage of anaerobic bacteria that is sensitive to it is high, and it is potent enough to include coverage of both aerobic and anaerobic infections as well. It has rapid bactericidal effects, penetrates the tissue well, and has a low risk of *Clostridioides difficile* colitis. Metronidazole also has been found to be effective in treating brain abscesses. While newer combination therapies have been developed for the treatment of mixed aerobic and anaerobic infection, there was, however, no clear advantage of these new combinations over metronidazole [2].

Metronidazole's most common side effects are diarrhea, abdominal pain, and nausea [3]. It has been reported in the literature that metronidazole can cause liver injury [4], neurotoxicity [5], pancreatitis [6], and possible mania [7]. It can also cause disulfiram-like reactions similar to other drugs like sulfonamides, nitrofurantoin, and chloramphenicol as they’re believed to interfere with alcohol metabolism causing these reactions that can be severe enough to cause mortality. It inhibits the oxidation of acetaldehyde leading to a buildup of high levels of acetaldehyde. Toxic acetaldehyde levels cause manifestations like flushing, nausea, vomiting, anxiety, headache, shortness of breath, tachycardia, palpitations, hypotension, and dysrhythmia. Acetaldehyde causes those manifestations through direct and indirect effects of histamine release [8-10]. While our patient had common symptoms as disulfiram-like reactions (nausea, dysrhythmia), his ethanol level was negative.

The drug-induced long QT interval is thought to be due to the blockade of delayed rectifier potassium channels (IKr) during the repolarization of the phase of the ventricular myocardium. This results in prolongation of cardiac action potential and afterdepolarization to be early. This causes the development of a second action potential which may demonstrate on ECG as a premature complex. This dysrhythmia can result in torsades de pointes (TdP) on ECG. The patient may be asymptomatic or alternatively present with ventricular tachycardia, palpations, syncope, or even sudden death [11]. Drug-induced QT interval prolongation occurs more frequently in patients that carry silent mutations in the genes associated with congenital QT syndromes. Exposure to drugs that block potassium channels in those patients puts them at high risk of developing TdP [12].

Some azole derivatives like ketoconazole, itraconazole, and fluconazole can result in TdP by their independent QT prolongation effect as well as their interactions with other QT-prolonging agents. Metronidazole is an azole derivative [13] that acts as an inhibitor of CYP3A4 and CYP2C9 resulting in QT-prolongation with its interactions with other QT prolongation agents. Furthermore, in a previous study, metronidazole was considered a cytochrome P-450 CYP3A4 inhibitor resulting in decreased clearance of quinine, a QT-prolonging agent [14].

The first reported case of metronidazole-induced QT interval prolongation was a 90-year-old lady that was given ceftriaxone and metronidazole. Her ECG demonstrated a QT interval of 324ms prior to medication administration and 703ms on the following day. Metronidazole was withdrawn and as a result her QT interval gradually returned to its initial value. This patient demonstrated that there is a possible link between metronidazole and QT prolongation. The QT prolongation could have been caused by factors other than metronidazole independently such as her age, co-morbidities, or drug interaction of metronidazole with ceftriaxone, a QT-prolonging drug [13,15].

Ventricular fibrillation is defined as a disturbance in the electrical activity of the cardiac tissue due to irregularity in the electrical waves. Therefore, the ventricular contraction doesn’t produce effective tension in the systole; hence, heart rate increases to allow for adequate blood circulation. Ventricular fibrillation is diagnosed on electrocardiography through the occurrence of irregular and aperiodic beat-to-beat changes during the electrical complexes of the ventricular [16].

Even with QT prolongation being linked to metronidazole in some cases, no report has linked metronidazole to ventricular fibrillation. Yet, our patient was not on any other medications and has no known co-morbidity.

Limitations

One of the main limitations that we recognize we’ve faced with this case report is the means by which this patient’s care was concluded. The patient fled the hospital without being noticed by the nursing staff, likely to escape his military training service. This led to a termination of further investigations and workup for the arrhythmia incident, which in turn may have impeded the accuracy of the reporting of this case.

Another restricting factor that we faced is the fact that the patient started giving different histories of presenting illness at different intervals. The initial story was that he wanted to end his life by overdosing on metronidazole on the morning of his presentation; however, on his communication with the MICU services (after psychiatry assessment), he noted only taking two tablets the night before. The contradicting stories affect the accuracy of this reporting. However, the rise in his liver functions in comparison to his presentation 14 days ago does suggest the possibility of a drug overdose, but not specific only to that.

A third limitation to add is the fact that we have no baseline ECG reading for the patient prior to this presentation, which occurred after the ingestion of metronidazole. This limits us from knowing whether or not the patient had an existing congenital QT syndrome which might have been a factor in this patient’s arrhythmia.

## Conclusions

The most likely theory for our patient having ventricular fibrillation is a consequence of TdP following prolongation of the QT interval prolongation. Metronidazole has been linked with QT prolongation, but it is not known to cause QT prolongation independently. However, the exact mechanism behind it remains unclear and needs further studies. To the best of our knowledge, our case is the only case that presents the possibility of metronidazole independently inducing ventricular fibrillation.
